# Cannabinoid receptor 2 deletion influences social memory and synaptic architecture in the hippocampus

**DOI:** 10.1038/s41598-021-96285-9

**Published:** 2021-08-19

**Authors:** Joanna Agnieszka Komorowska-Müller, Kishore Aravind Ravichandran, Andreas Zimmer, Britta Schürmann

**Affiliations:** Institute for Molecular Psychiatry, Medical Faculty, University Hospital Bonn, University of Bonn, Venusberg-Campus 1, Bldg. 76, 53127 Bonn, Germany

**Keywords:** Learning and memory, Molecular neuroscience, Synaptic transmission

## Abstract

Although the cannabinoid receptor 2 (CB_2_R) is often thought to play a role mainly outside the brain several publications unequivocally showed the presence of CB_2_R on hippocampal principal neurons. Activation of CB_2_R produced a long-lasting membrane potential hyperpolarization, altered the input/output function of CA2/3 principal neurons and produced alterations in gamma oscillations. However, other cellular, molecular and behavioral consequences of hippocampal CB_2_R signaling have not been studied in detail. Here we demonstrate that the deletion of CB_2_ leads to a highly significant increase in hippocampal synapsin-I expression levels and particle density, as well as increased vesicular GABA transporter (vGAT) levels. This phenotype was restricted to females and not observed in males. Furthermore, we demonstrate an impairment of social memory in CB_2_ deficient mice. Our results thus demonstrate that the lack of CB_2_R leads to changes in the hippocampal synaptic landscape and reveals an important sex-specific difference in endocannabinoid signaling. This study supports a significant role of the CB_2_R in modulation of different types of memory despite its low expression levels in the brain and provides more insight into a sex-specific role of CB_2_R in synaptic architecture.

## Introduction

The endocannabinoid system (ECS) consists of endocannabinoids, their receptors, and their synthesizing and degrading enzymes. The two main cannabinoid receptors are: cannabinoid receptor 1 (CB_1_R) and cannabinoid receptor 2 (CB_2_R, encoded by the gene *Cnr2*). CB_1_R has been extensively studied in the brain, whereas CB_2_R for a long time has been regarded as the peripheral cannabinoid receptor, present mostly on endocrine and immune cells^1,2^. Its characterization in the brain has been a challenge due to its low baseline expression and a lack of reliable antibodies^3^. Initially, CB_2_R was detected in the brain during pathological conditions including Alzheimer’s disease and chronic pain due to an upregulation in immune cells of the brain–microglia^4–7^.

Recent technological advances in the field of molecular biology brought compelling evidence for neuronal *Cnr2* expression. Using RNAScope^8^, *Cnr2* was detected on excitatory and inhibitory neurons in the hippocampus^9,10^. Furthermore, neuronal *Cnr2* expression has been found in the ventral tegmental area, prefrontal cortex, cerebellum, dorsal striatum and nucleus accumbens^11–13^.

Acute activation of brain CB_2_R reduces inhibitory synaptic transmission in the rat medial entorhinal area and neuronal excitability in the hippocampus^9,14^, whereas chronic activation increases excitatory synaptic transmission^15^. The constitutive deletion of CB_2_R results in decreased excitatory synaptic transmission and a reduced magnitude of long-term potentiation^9,16–19^. Furthermore, stimulation of CB_2_R in CA2/3 hippocampal neurons, but not CA1 or dentate gyrus neurons, elicits a long-lasting hyperpolarization^9^. This hyperpolarization in the hippocampus is strictly CB_2_R dependent—it can be blocked with CB_2_R antagonists, can be elicited with CB_2_R agonist and is absent in CB_2_R knockout animals. Additionally, the hyperpolarization appears to be a cell-intrinsic self-regulatory mechanism that acts complementary to presynaptic CB_1_Rs. On a network level systemic treatment with CB_2_R agonist results in a decreased modulation of slow-gamma oscillation dependent on the theta oscillation amplitude in area CA3^9^. These results imply that the CB_2_R-dependent hyperpolarization regulates hippocampal network activity.

However, it is unknown if this very specific form of CB_2_R-dependent plasticity affects synaptic architecture in CA2/3 area of the hippocampus. In this study, we addressed this question using CB_2_R-deficient mice. We show that CB_2_R deletion caused a sex-specific increase in synaptic proteins in the hippocampus and altered social memory. Thereby, our results provide evidence for an important role of CB_2_R in social memory function and synaptic architecture.

## Results

### Increased synapsin-I levels and puncta size in the hippocampus after CB_2_R deletion in females, but not in males

**Figure 1:**
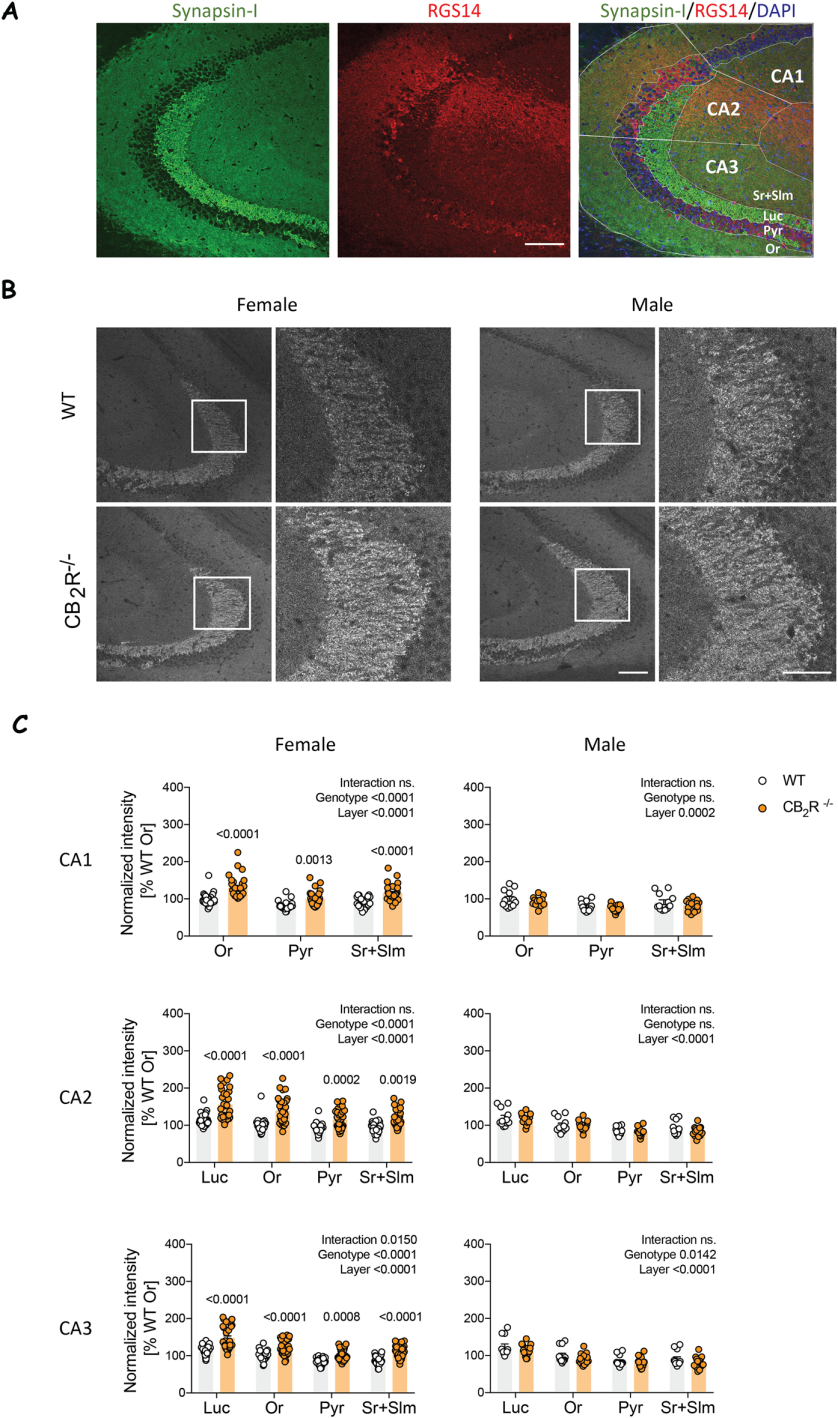
Synapsin-I levels are increased in the hippocampus after CB_2_R deletion in female mice. (**A**) Example images show synapsin-I (green), RGS14 (red) and DAPI (blue). White lines indicate manual delineation of CA regions and layers in the hippocampus. Scale bar: 100 µm. (**B**) Example images of synapsin-I immunoreactivity in male and female from WT and CB_2_R^−/−^ mice. The white box within the left panel indicates the section that is displayed in higher magnification on the right. Scale bar: 100 µm, 50 µm (inset). (**C**) Quantification of synapsin-I mean grey value within each hippocampal layer normalized to the result from stratum oriens (Or). *Or* stratum oriens, *Luc* stratum lucidum, *Pyr* stratum pyramidale, *Sr + Slm* stratum radiatum and stratum lacunosum-moleculare. White circle—WT; orange circle—CB_2_R^−/−^ mice. Each datapoint represents one image. Left panel—female mice (WT: n = 30 images from N = 9 mice; CB_2_R^−/−^: n = 31 images from N = 9 mice); right panel—male mice (WT: n = 12 images from N = 3 mice; CB_2_R^−/−^: n = 16 images from N = 4 mice). Top panel—CA1 region; middle panel—CA2 region; bottom panel—CA3 region. Data is displayed as mean value $\pm$ SEM. Data was analyzed by two-way ANOVA followed by Sidak’s multiple comparison test. The exact p-values are indicated on the graph and reported in Supplementary Table S1.

To assess general changes in synapse density and size in the hippocampus we investigated synapsin-I immunostaining in the CA1, CA2 and CA3 area. The areas were discriminated based on a staining with an antibody against RGS14, a marker for CA2 principal cells (Fig. 1A). First, we analyzed the mean grey value in all hippocampal areas (Fig. 1B). We identified a significant sex and sex × genotype interaction effect and therefore analyzed females and males individually (Supplementary Table S2). As expected, we detected a significant effect of hippocampal layer in all areas with the highest density of synpasin-I in stratum lucidum. Furthermore, we found a significant genotype effect in females in all areas of the hippocampus (Fig. 1C). Posthoc analysis revealed significantly increased synapsin-I levels in all areas and all layers. In male mice, a genotype effect was present in the CA3 area, but posthoc analysis did not reveal any layer-specific genotype difference.

**Figure 2:**
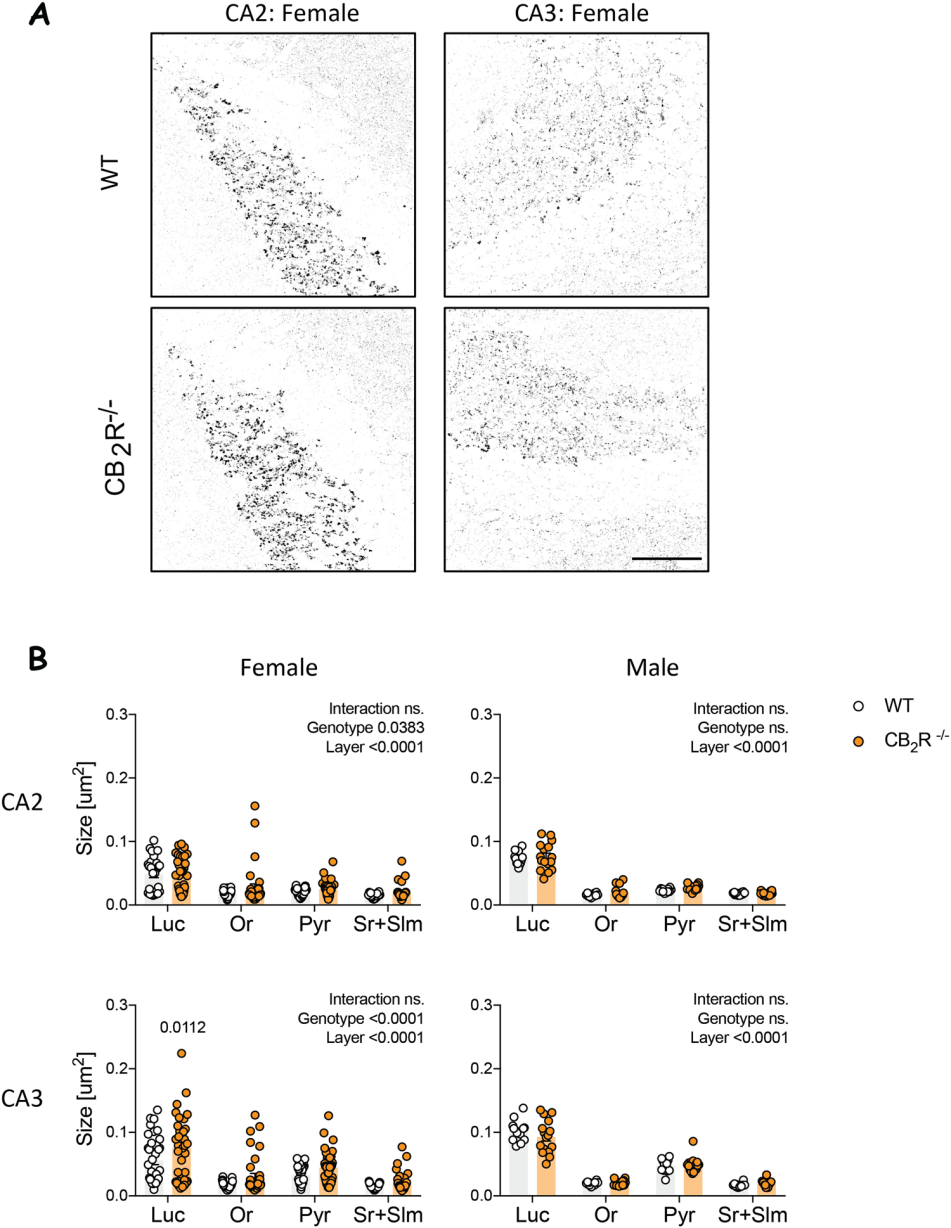
Average size of synapsin-I particles is increased after CB_2_R deletion in females. (**A**) Example images show binary image of synapsin-I. Scale bar: 50 µm. (**B**) Quantification of synapsin-I mean puncta size (detected puncta > 0.005 µm). *Or* stratum oriens, *Luc* stratum lucidum, *Pyr* stratum pyramidale, *Sr + Slm* stratum radiatum and stratum lacunosum-moleculare. White circle—WT; orange circle—CB_2_R^−/−^ mice. Each datapoint represents one image. Left panel—female mice (WT: n = 28–35 images from N = 9 mice; CB_2_R^−/−^: n = 31–36 images from N = 9 mice); right panel—male mice (WT: n = 12 images from N = 3 mice; CB_2_R^−/−^: n = 16 images from N = 4 mice). Top panel—CA2 region; bottom panel—CA3 region. Data is displayed as mean value $\pm$ SEM. Data was analyzed by two-way ANOVA followed by Sidak’s multiple comparison test. The exact p-values are indicated on the graph and reported in Supplementary Table S1.

To address if the increased synapsin-I signal in females was due to an increase in the size of synaptic clusters, we analyzed the average size of synapsin-I puncta specifically in the CA2 and CA3 area (Fig. 2A). Our analysis showed an overall genotype effect in both CA2 and CA3, exclusively in females (Fig. 2B). The following posthoc analysis did not reveal any specific changes in any of the CA2 layers. However, we observed a significant increase in stratum lucidum of the CA3 hippocampal area. In contrast, no genotype effect was present in male mice.

Furthermore, we detected a layer effect in both males and females in the CA2 and CA3 area of the hippocampus with the biggest puncta size in stratum lucidum (Fig. 2B).

### CB_2_R deletion alters excitatory and inhibitory synapses in a sex-specific manner

**Figure 3:**
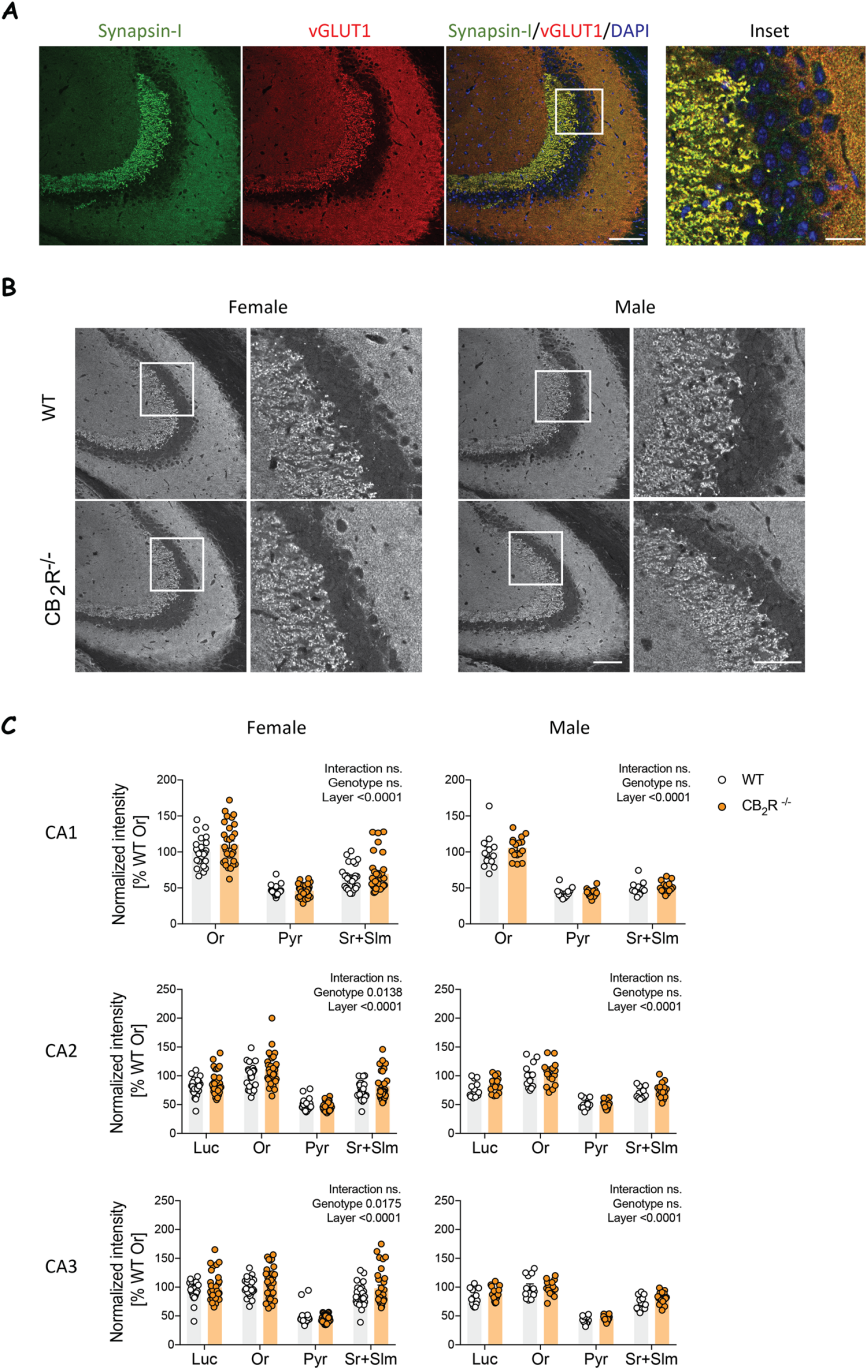
vGLUT1 levels are slightly increased in female CB_2_R^−/−^ mice. (**A**) Example images show synapsin-I (green), vGLUT1 (red) and DAPI (blue). Scale bar: 100 µm, 25 µm (inset). (**B**) Example images of vGLUT1 immunoreactivity in male and female from WT and CB_2_R^−/−^ mice. The white box within the left panel indicates the section that is displayed in higher magnification on the right. Scale bar: 100 µm, 50 µm (inset). (**C**) Quantification of vGLUT1 mean grey value within each hippocampal layer normalized to the result from stratum oriens (Or). *Or* stratum oriens, *Luc* stratum lucidum, *Pyr* stratum pyramidale, *Sr + Slm* stratum radiatum and stratum lacunosum-moleculare. White circle—WT; orange circle—CB_2_R^−/−^ mice. Each datapoint represents one image. Left panel—female mice (WT: n = 30 images from N = 9 mice; CB_2_R^−/−^: n = 31 images from N = 9 mice); right panel—male mice (WT: n = 12 images from N = 3 mice; CB_2_R^−/−^: n = 16 images from N = 4 mice). Top panel—CA1 region; middle panel—CA2 region; bottom panel—CA3 region. Data is displayed as mean value $\pm$ SEM. Data was analyzed by two-way ANOVA followed by Sidak’s multiple comparison test. The exact p-values are indicated on the graph and reported in Supplementary Table S1.

We then investigated excitatory synapses in the CA areas of the hippocampus using vesicular glutamate transporter 1 (vGLUT1) as a marker. Co-staining with vGLUT1 and synapsin-I revealed a high degree of colocalization between those markers (Fig. 3A). As for synapsin-I, we found an overall layer effect and a main genotype effect in the CA2 and CA3 areas in female mice (Fig. 3B,C). However, we detected no genotype effect in the CA1 region.

The subsequent posthoc analysis in CA2 and CA3 did not reveal any layer-specific effect. In males, there was no genotype effect. Our data indicate that vGLUT1 expression was altered in female CB_2_R^−/−^ mice, but the effects were not as pronounced as the changes observed for synapsin-I.

**Figure 4:**
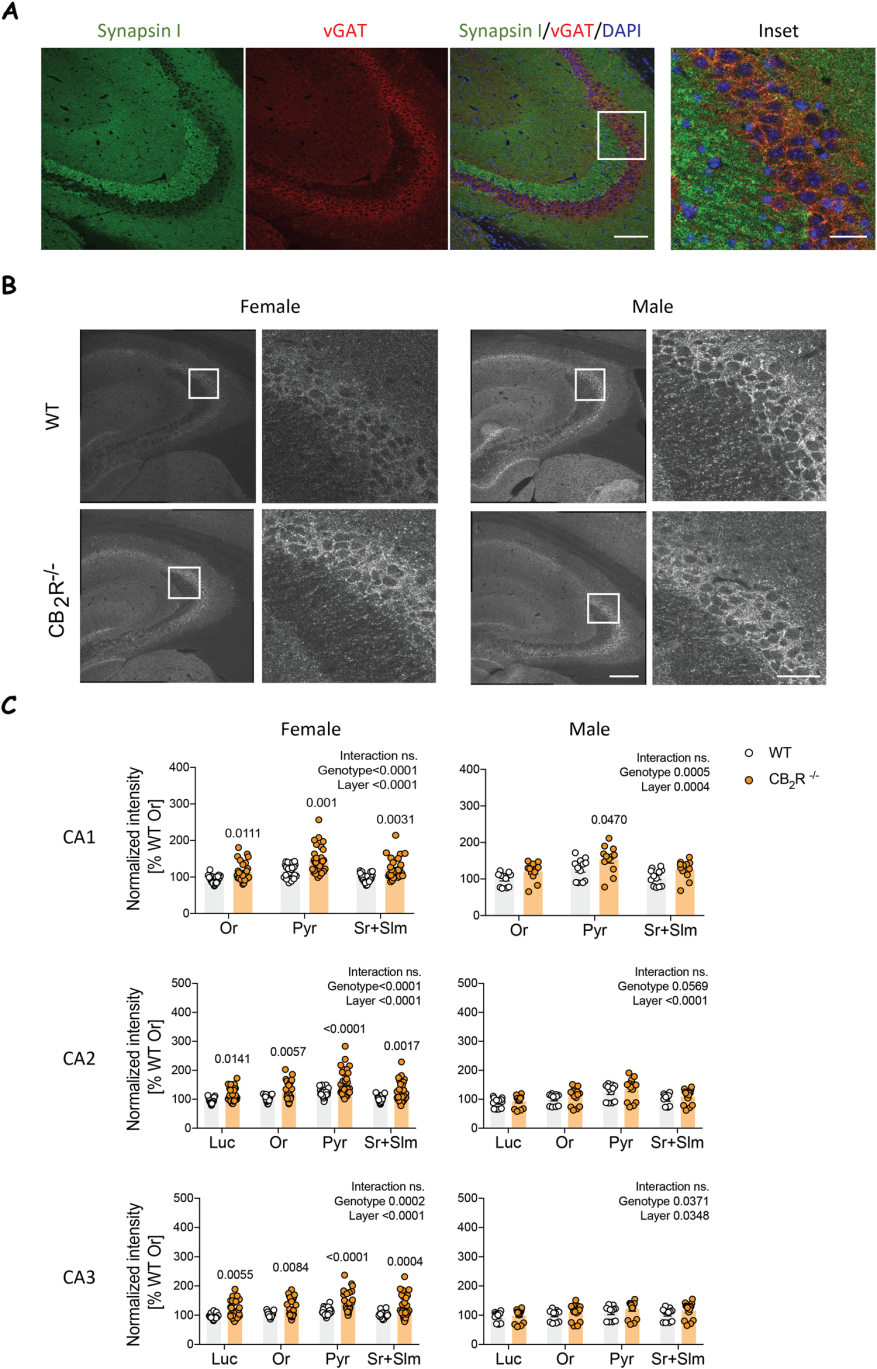
vGAT levels are increased in female CB_2_R^−/−^ mice. (**A**) Example images show synapsin-I (green), vGAT (red) and DAPI (blue). Scale bar: 100 µm, 25 µm (inset). (**B**) Example images of vGAT immunoreactivity in male and female from WT and CB_2_R^−/−^ mice. The white box within the left panel indicates the section that is displayed in higher magnification on the right. Scale bar: 200 µm, 25 µm (inset). (**C**) Quantification of vGAT mean grey value within each hippocampal layer normalized to the result from stratum oriens (Or). *Or* stratum oriens, *Luc* stratum lucidum, *Pyr* stratum pyramidale, *Sr + Slm* stratum radiatum and stratum lacunosum-moleculare. White circle—WT; orange circle—CB_2_R^−/−^ mice. Each datapoint represents one image. Left panel—female mice (WT: n = 30 images from N = 9 mice; CB_2_R^−/−^: n = 31 images from N = 9 mice); right panel—male mice (WT: n = 12 images from N = 3 mice; CB_2_R^−/−^: n = 16 images from N = 4 mice). Top panel—CA1 region; middle panel—CA2 region; bottom panel—CA3 region. Data is displayed as mean value $\pm$ SEM. Data was analyzed by two-way ANOVA followed by Sidak’s multiple comparison test. The exact p-values are indicated on the graph and reported in Supplementary Table S1.

We next investigated inhibitory synapses in the hippocampus using vGAT as a marker. The extent of colocalization between synapsin-I and vGAT staining is lower when compared with what we observed for synpasin-I and vGLUT1 (Fig. 4A). Consistent with the other synaptic markers we analyzed, we found a significant layer effect across all groups. Furthermore, we detected a main effect of the genotype in both female and male mice (Fig. 4B,C). Although an increase in vGAT mean gray values was observed in both sexes of CB_2_ knockout mice, it was again much more pronounced in females. A significant genotype effect was present for all areas and regions after posthoc analysis in female mice. However, male mice exhibited statistical significance only in CA1 stratum pyramidale.

### CB_2_R deletion decreases social memory in both male and female mice

Our synaptic marker analysis showed the most prominent changes in the CA2/3 areas. The CA2 hippocampal area is important for social memory^20^. Hence, we investigated short-term social memory in CB_2_R^−/−^ mice using the partner recognition test (PR).

**Figure 5:**
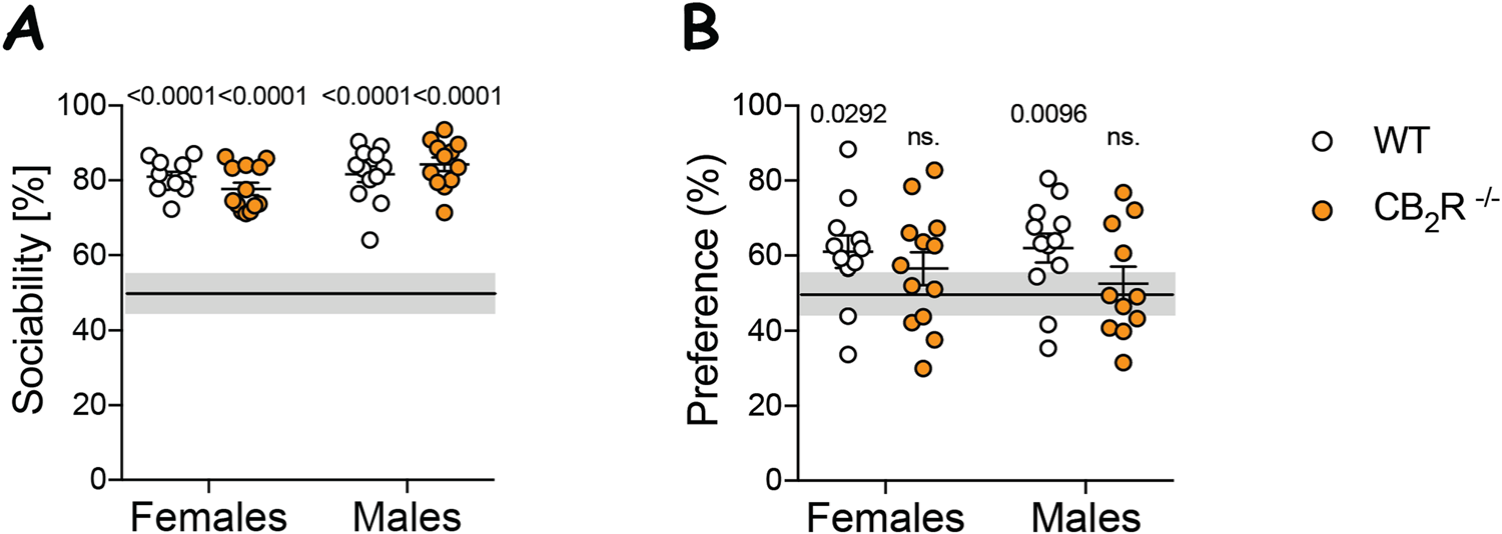
CB_2_R deletion decreases social memory in both male and female mice. (**A**) Sociability (%) was calculated as interaction time with a partner mouse over total interaction time. A mean value above 50% indicates that the mice are social. (**B**) Preference (%) for the novel partner was calculated as time with the novel partner mouse over total interaction time. Significant difference from the chance level (50%) indicates learning. White circle—WT; orange circle—CB_2_R^−/−^ mice. Squares represent data from individual mice (N = 11–13 mice/sex/genotype). Data are displayed as mean value $\pm$ SEM. Each group was analyzed individually by one-sample t-test (hypothetical mean = 50). The exact p-values are indicated on the graph.

CB_2_R^−/−^ mice and controls displayed similar social behavior as indicated by a sociability above the 50% chance level (Fig. 5A). However, only WT mice preferred to interact with a novel partner, indicating that they remembered the familiar mouse from the previous trial (Fig. 5B). In contrast, for CB_2_R^−/−^ mice the preference for the novel partner did not differ from chance level, indicating a social memory impairment in these mice (Fig. 5B).

To investigate if this genotype effect was due to a sensory defect, we performed an olfaction test. Mice from both genotypes showed a significant increase in the exploration of a urine-stained cotton swab over a water-stained swab (Supplementary Fig. S2). Overall, these data suggest that CB_2_R^−/−^ impairs social memory in the PR test in both male and female mice.

## Discussion

The results from this study show that deletion of CB_2_R alters the synaptic architecture in the hippocampus and results in social memory deficit. Thus, despite the low level of expression of CB_2_Rs in neurons, their signaling plays an important role in synaptic functions. Moreover, our results reveal a pronounced sex effect, with female mice showing highly significant alterations in expression of hippocampal synaptic markers, in contrast to male mice. Our findings thus add to the growing evidence of important sex-specific differences in endocannabinoid signaling^21^.

Although reports indicating an important role of CB_2_R in the modulation of neuronal functions were initially met with skepticism in the research community, the accumulated evidence in arguing for such a role is now compelling. For instance, acute activation of CB_2_R decreased neuronal firing frequency and reduced the excitability of neurons, whereas chronic activation leads to increased neuronal excitability^9,19^. At the same time, CB_2_R has been implicated in higher cognitive functions, as CB_2_R deletion led to alterations in memory performance^7,22–24^, with a global CB_2_R deletion impairing aversive memory, but enhancing working memory^22,24,25^.

On a cellular level, CB_2_R deletion leads to a decreased excitatory transmission and decreased dendritic spine numbers in the hippocampus^18,26^. To further investigate synaptic changes in the hippocampus after CB_2_R deletion, we stained for a general presynaptic marker—synapsin-I and observed an increase in synapsin-I levels exclusively in CB_2_R^−/−^ female mice. The change in synapsin-I intensity was accompanied by an increase in synapsin-I average puncta size in both CA2 and CA3 area of the hippocampus. It is possible that an increase in the presynaptic vesicle pool is a compensatory mechanism to counteract the decreased number of dendritic spines. On the other hand, synapsins have been shown to boost synaptic release at high firing rates by recruiting more vesicles from the reserve vesicle pool at excitatory synapses^27^. Whether the observed increase comes from an increase in overall vesicle number or an alteration in synaptic facilitation should be addressed in the future. In addition, loss of synapysin-I decreases baseline transmission at inhibitory synapses^27^. In agreement with that, decreasing synapsin-I expression also decreases the pool of readily releasable synaptic vesicles and alters short-term synaptic plasticity, while not affecting memory or long-term potentiation in young mice^28–31^. Thus, an increase in synapsin-I could explain the improved working memory in CB_2_R^−/−^ mice^24^.

Alterations in synapsins are known to affect both excitatory and inhibitory synapses although in a different capacity. Furthermore, *Cnr2* has been detected on both excitatory and inhibitory cells^10^. Following the discovery of increased synapsin-I levels in females, we asked if the increase in presynaptic vesicle staining was due to alterations in glutamatergic or GABAergic synapses. Both vGLUT1 and vGAT intensities were enhanced in CB_2_R^−/−^ female mice, suggesting that both excitatory and inhibitory synapses are regulated by CB_2_R.

We observed a stronger effect for inhibitory synapses. It is known that even subtle alterations in perisomatic inhibitory synapses go along with perturbations in gamma oscillations^32^. Thus, our findings might explain the observed CB_2_R-dependent alterations of gamma oscillations^9^. However, we do see changes in both excitatory and inhibitory synapses and those changes seem to be prominent in all hippocampal layers and most hippocampal regions. Therefore, it is likely that these marker proteins are not directly regulated by CB_2_R activity but rather reflect an overall change in the synaptic landscape of the hippocampus. Furthermore, our data indicate that CB_2_R modulates hippocampal networks differently in males and females.

In light of these sex-specific phenotypes, it is important to note that most of the previous studies performed on CB_2_R knockouts were done with sex-mixed groups^7,24,33–35^ or exclusively with male mice^18,22,25,36–38^. It is therefore possible that sex-specific phenotypes have been overlooked and that the relevance for CB_2_R-dependent processes in the central nervous system have been underestimated. It is not clear why females have a stronger CB_2_R knockout phenotype, but these sex differences may be related to differential CB_2_R expression levels. It has been already shown that *Cnr2* expression in the hippocampus, prefrontal cortex and hypothalamus is higher in female than in male mice^39^.

We also evaluated social behavior and social memory in this study because Stempel et al. described a CB_2_R-dependent hyperpolarization phenomenon in CA2/3 area^9^. The CA2 area in particular has been implicated in social memory^20^. Indeed, we observed that short-term social memory was impaired in both female and male CB_2_R^−/−^ mice. Further studies deleting CB_2_R specifically in the CA2 region, are necessary to elucidate CB_2_R functions in hippocampal neurocircuits in more detail. Such studies are important because the pronounced cognitive phenotype after CB_2_R deletion now clearly reveals a significant role of CB_2_Rs in the modulation of different forms of memory, despite its low expression levels.

## Materials and methods

### Animals

The experiments were carried out with 4–6-month old female and male mice. Food and water were provided ad libitum. To investigate the results of the constitutive *Cnr2* deletion, we compared B6.Cg-*Cnr2*^tm1Zim^ (CB_2_R^−/−^; MGI Cat# 2663848, RRID: MGI:2663848)^40^ with C57BL6/J (WT) mice. CB_2_R^−/−^ were generated using homologous recombination and have a constitutive 131 amino acid deletion at the C-terminus of the CB_2_R eliminating part of the intracellular loop 3 and transmembrane domains 6 and 7^40^. Among others, CB_2_R^−/−^ have impaired neuromodulatory functions, accelerated age-dependent bone loss and obesity^40–42^. Recently, CB_2_R^−/−^ mice were also reported to have aversive memory impairment^22,24,25^.

Care of the animals and conduction of the experiments followed the guidelines of the European Communities Directive 86/609/EEC and the German Animal Protection Law regulating animal research and were approved by the ethics committee of the Landesamt für Natur, Umwelt und Verbraucherschutz Nordrhein-Westfalen (LANUV NRW), Germany (AZ 84-02.04.2017.A231). The experiments were carried out in accordance with ARRIVE guidelines. Independent groups were used for behavioral testing and immunohistochemical analysis. For behavioral testing, an independent male and female groups were used (N = 11–13 mice/group). For immunohistological analysis two independent groups of mice were used. One group included male (N = 3–4 mice/genotype) and female (N = 4 mice/genotype) mice from WT and CB_2_R^−/−^. Another group included only female mice (N = 4–5 mice/genotype).

### Behavioral testing

A male and a female group were tested independently with 11–13 mice/sex/genotype. The experimenter was blind with regards to the genotype. A week before the first test, mice were single-housed and transferred to a room with a reversed light–dark cycle (lights off at 9:00 a.m.). Behavioral tests were conducted during the dark phase (from around 9:30 a. m.).

#### Partner recognition test (PR)

This paradigm was used to asses social memory. Animal activity was recorded with the EthoVision XT 13 (Noldus, RRID:SCR_000441). The test was performed in an open-field box (44 cm × 44 cm) containing a 1 cm layer of sawdust under dim illumonation. For three consecutive days, mice were allowed to explore the arena freely for 10 min and were habituated to the environment. On the test day, mice underwent two trials. In trial 1, mice were given 9 min to freely explore the arena containing an object (metal can) and a grid cage (diameter around 10 cm, height around 12 cm) with an unfamiliar C57BL6/J male partner mouse. The can and the cage were in opposite corners, each placed around 6–7 cm from the wall. Partner mice were approximately 10 weeks old. Interaction was noted when the mouse nose point was within 2 cm of the cage/can. The time spent on top of any of the objects was deduced from the interaction time. After trial 1, mice were put back to their homecages for 1 h. Sociability in trial 1 was calculated as follows: sociability (%) = Tp/(Tp + Tc) × 100, where Tp is the time of interaction with a partner mouse and Tc is the time of interaction with the object. To detect if the sociability of the group was greater than the chance level, the mean sociability value was tested with a one-sample t-test against a hypothetical mean (50%) representing the chance level. Sociability above 50% indicated that the mouse spent more time interacting with a partner than with an object. In trial 2, the metal can was replaced by a grid cage with a novel partner mouse and the test mouse was given 3 min to freely explore and interact with both partners. Preference for the novel partner was calculated as preference (%) = Tn/(Tf + Tn) × 100, where Tf is the time spent with the familiar partner and Tn is the time spent with a novel partner mouse. If the mouse recognized its previous partner, it should have spent > 50% of interaction time interacting with a novel partner. Therefore, a preference for the new partner is interpreted as learning. To detect learning in each group, the preference for the novel partner was tested with a one-sample t-test against a hypothetical mean (50%) to detect if it statistically deviates from the chance level.

### Brain isolation

Two independent cohorts of mice were transcardially perfused with phosphate-buffered saline (PBS). Brains were isolated and hemisected. One of the hemispheres was postfixed in 4% formaldehyde (pH = 6.9; Sigma- Aldrich) for 3.5–4 h on ice, shaking. They were cryoprotected using overnight incubation in 10% sucrose followed by an overnight incubation in 30% sucrose. Brains were frozen in dry ice-cooled isopentane and stored at − 80 °C until sectioning.

### Immunohistochemistry

Frozen-fixed hemispheres were cut with a cryostat (Microm HM500) into 20 μm sections and stored at − 20 °C. Prior to the staining, slices were thawed on a 37 °C heating plate for 30 min. They were then permeabilized for 10 min in 0.3% Triton X-100 in Tris- buffered saline (1 M TBS, pH = 7.5), followed by 3 × 5 min wash in TBS. Then, antigen retrieval in citrate buffer pH = 6 for 20 min at 65 °C was performed. The slides were washed 3 times with TBS and blocked for 1 h in blocking buffer (2% BSA, 10% normal goat serum, 0.3% Triton X-100 in TBS). Afterwards, the slides were incubated overnight with primary antibodies in blocking buffer: 1:250 rabbit anti-synapsin-I (Abcam Cat# ab64581, RRID:AB_1281135), 1:1000 guinea pig anti-vGLUT1 (Millipore Cat# AB5905, RRID:AB_2301751), 1:200 guinea pig anti-vGAT (Synaptic Systems Cat# 131 004, RRID:AB_887873), 1:5 mouse anti-RGS14 (Antibodies Incorporated Cat# 73-170, RRID:AB_10698026). The next day, the slides were incubated for 20 min at room temperature and washed 3 times in TBS. Then they were blocked for 1 h followed by an incubation with 1:1000 AlexaFluor^®^ conjugated secondary antibodies in blocking solution: goat anti-mouse AF568 (Molecular Probes Cat# A-11031, RRID:AB_144696), goat anti-rabbit AF488 (Molecular Probes Cat# A-11008, RRID:AB_143165), goat anti-guinea pig AF647 (Abcam Cat# ab150187, RRID:AB_2827756). Afterwards, the slides were washed 3 times in TBS, stained for 4′,6-Diamidin-2-phenylindol (DAPI), washed once in TBS and mounted using ProLong™ Diamond Antifade Mountant (Life technologies, P36961).

### Image acquisition and analysis

The experimenter was blind with regards to the genotype during image acquisition and analysis. Single-plane confocal pictures were obtained with a LeicaSP8 inverted confocal microscope equipped with a 20× water immersion objective (N.A. 0.75). For synaptic quantification high-magnification single-plane confocal images of hippocampal areas were obtained using a LeicaSP8 inverted confocal microscope and a 63× water immersion objective (N.A. 1.2). The imaging conditions were defined once for each staining and kept during all imaging sessions. For presentation, images were post-processed in ImageJ to adjust brightness and contrast. For quantification, images were analyzed using ImageJ. All regions of interest (ROI) were delineated manually using RGS14/DAPI/synapsin-I merged image. In 20× and 63× images, the mean grey value was measured within each ROI. For particle analysis images were thresholded using a fixed mean grey value threshold to obtain a binary image. An appropriate threshold per staining was determined as a mean of manual thresholds set for all the pictures. The same threshold was applied to all images and genotypes within a staining. The number and average size of puncta per ROI was counted using Image J’s 'Analyze Particles' function (minimum puncta size: 0.005 μm). For each animal 3–4 images were taken. Data were normalized as % of mean of control group (stratum oriens). Data from immunohistological groups were pooled together per sex. Mean per animal was included in Supplementary Fig. S1 and the corresponding statistical analysis in Supplementary Table 3.

### Statistical analysis and data presentation

Microsoft Excel was used for data analysis followed by statistical analysis and data visualization in GraphPad Prism version 7.0.0 and 9.1.2 for Mac, GraphPad Software, San Diego, California USA, www.graphpad.com. Figures were put together in Adobe Illustrator 2020. Behavioral data analysis was done using EthoVision XT 13 (Noldus, RRID:SCR_000441). For immunohistological data two-way ANOVA was used (independent variables: genotype and layer) followed by Sidak’s multiple comparison test. To identify differences between sexes in Synapsin-I levels, three-way ANOVA was used (independent variables: genotype, sex and layer; Supplementary Table S2). For PR, one-sample t-test was used to test the mean of the group against a theoretical mean of 50 to detect significant difference of each group from the chance level. Statistical significance was stated when p-value < 0.05 at a 95% confidence interval.

## Supplementary Information

Supplementary Information.

## Data Availability

Datasets are available on request. The raw data supporting the conclusions of this article will be made available by the authors, without undue reservation, to any qualified researcher.
