# Supplementary material for: Cannabinoid receptor 2 deletion influences social memory and synaptic architecture in the hippocampus
Source: Sci Rep. 2021 Aug 19;11:16828. doi: 10.1038/s41598-021-96285-9 (PMC8376893; doi:10.1038/s41598-021-96285-9)
Supplement: Supplementary file 1 — Supplementary Information. [file 41598_2021_96285_MOESM1_ESM.pdf]

Supplementary Table S1. Detailed results from 2-way ANOVA analysis.

		Statistical test		Posthoc test	
Figure	Panel	Test name	result	Test name	result
1. Synapsin-I levels are increased in the hippocampus after CB2R deletion in female mice	**1c:** females: CA1	2-way ANOVA	layer "F (2, 171) = 25.16", p < 0.0001; genotype "F (1, 171) = 64.42", p < 0.0001	Šídák's multiple comparisons test	Or p < 0.00001; Pyr p = 0.0013; Sr+Slm p <0.0001
1. Synapsin-I levels are increased in the hippocampus after CB2R deletion in female mice	**1c:** males: CA1	2-way ANOVA	layer "F (2, 75) = 9.820", p = 0.0002		
1. Synapsin-I levels are increased in the hippocampus after CB2R deletion in female mice	**1c:** females: CA2	2-way ANOVA	layer "F (3, 236) = 22.06", p < 0.0001; genotype "F (1, 236) = 102.4", p < 0.0001	Šídák's multiple comparisons test	Luc p < 0.0001; Or p < 0.00001; Pyr p = 0.0002; Sr+Slm p = 0.0019
1. Synapsin-I levels are increased in the hippocampus after CB2R deletion in female mice	**1c:** males: CA2	2-way ANOVA	layer "F (3, 104) = 27.58", p < 0.0001		
1. Synapsin-I levels are increased in the hippocampus after CB2R deletion in female mice	**1c:** females: CA3	2-way ANOVA	layer "F (3, 236) = 51.37", p < 0.0001; genotype "F (1, 236) = 110.2", p < 0.0001; layer x genotype "F (3, 236) = 3.557", p = P=0.0150	Šídák's multiple comparisons test	Luc p < 0.0001; Or p < 0.00001; Pyr p = 0.0008; Sr+Slm p <0.0001
1. Synapsin-I levels are increased in the hippocampus after CB2R deletion in female mice	**1c:** males: CA3	2-way ANOVA	layer "F (3, 104) = 27.58", p < 0.0001		
2. Average size of Synapsin-I particles is increased after CB2R deletion in females.	**2b:** females: CA2	2-way ANOVA	layer "F (3, 228) = 31.96", p < 0.0001; genotype "F (1, 228) = 4.343", p = 0.0383	Šídák's multiple comparisons test	-
2. Average size of Synapsin-I particles is increased after CB2R deletion in females.	**2b:** males: CA2	2-way ANOVA	layer "F (3, 104) = 214.0", p < 0.0001		
2. Average size of Synapsin-I particles is increased after CB2R deletion in females.	**2b:** females: CA3	2-way ANOVA	layer "F (3, 276) = 43.86", p < 0.0001; genotype "F (1, 276) = 16.07", p < 0.0001	Šídák's multiple comparisons test	Luc p = 0.0112
2. Average size of Synapsin-I particles is increased after CB2R deletion in females.	**2b:** males: CA3	2-way ANOVA	layer "F (3, 104) = 220.4", p < 0.0001		
3. vGLUT1 levels are slightly increased in female CB2R-/- mice.	**3c:** females: CA1	2-way ANOVA	layer "F (2, 162) = 130.6", p < 0.0001		
3. vGLUT1 levels are slightly increased in female CB2R-/- mice.	**3c:** males: CA1	2-way ANOVA	layer "F (2, 78) = 172.2", p < 0.0001		
3. vGLUT1 levels are slightly increased in female CB2R-/- mice.	**3c:** females: CA2	2-way ANOVA	layer "F (3, 224) = 94.36", p < 0.0001; genotype "F (1, 224) = 6.166", p = 0.0138	Šídák's multiple comparisons test	-
3. vGLUT1 levels are slightly increased in female CB2R-/- mice.	**3c:** males: CA2	2-way ANOVA	layer "F (3, 104) = 61.08", p < 0.0001		
3. vGLUT1 levels are slightly increased in female CB2R-/- mice.	**3c:** females: CA3	2-way ANOVA	layer "F (3, 224) = 91.73", p < 0.0001; genotype "F (1, 224) = 5.725", p = 0.0175	Šídák's multiple comparisons test	-
3. vGLUT1 levels are slightly increased in female CB2R-/- mice.	**3c:** males: CA3	2-way ANOVA	layer "F (3, 104) = 104.0", p < 0.0001		
4. vGAT levels are increased in female CB2R-/- mice, but not in male.	**4c:** females: CA1	2-way ANOVA	layer "F (2, 155) = 18.93", p < 0.0001; genotype "F (1, 155) = 36.59", p < 0.0001	Šídák's multiple comparisons test	Or p = 0.0111; Pyr p = 0.0001; Sr+Slm p = 0.0031
4. vGAT levels are increased in female CB2R-/- mice, but not in male.	**4c:** males: CA1	2-way ANOVA	layer "F (2, 66) = 8.762", p = 0.0004; genotype "F (1, 66) = 13.55", p = 0.0005	Šídák's multiple comparisons test	Pyr p = 0.0470
4. vGAT levels are increased in female CB2R-/- mice, but not in male.	**4c:** females: CA2	2-way ANOVA	layer "F (3, 216) = 17.29", p < 0.0001; genotype "F (1, 216) = 55.13", p < 0.0001	Šídák's multiple comparisons test	Luc p = 0.0141; Or p = 0.0057; Pyr p <0.0001; Sr+Slm p = 0.0017
4. vGAT levels are increased in female CB2R-/- mice, but not in male.	**4c:** males: CA2	2-way ANOVA	layer "F (3, 100) = 11.19", p < 0.0001; genotype "F (1, 100) = 3.711", p = 0.0569		
4. vGAT levels are increased in female CB2R-/- mice, but not in male.	**4c:** females: CA3	2-way ANOVA	layer "F (3, 216) = 6.922", p = 0.0002; genotype "F (1, 216) = 53.83", p < 0.0001	Šídák's multiple comparisons test	Luc p = 0.0055; Or p = 0.0084; Pyr p <0.0001; Sr+Slm p = 0.0004
4. vGAT levels are increased in female CB2R-/- mice, but not in male.	**4c:** males: CA3	2-way ANOVA	layer "F (3, 100) = 2.986", p = 0.0348; genotype "F (1, 100) = 4.463", p = 0.0371		

Supplementary Table S2. Detailed results from 3-way ANOVA analysis.

		Statistical test	
Dateset	Area	Test name	result
Synapsin-I levels in the hippocampus	**CA1**	3-way ANOVA	layer "F (2, 159) = 20.80", p < 0.0001; genotype "F (1, 159) = 15.83", p = 0.0001; sex "F (1, 159) = 26.71", p < 0.0001; genotype x sex "F (1, 159) = 42.42", p < 0.0001
	**CA2**	3-way ANOVA	layer "F (3, 224) = 53.35", p < 0.0001; genotype "F (1, 224) = 70.77", p < 0.0001; sex "F (1, 224) = 41.64", p < 0.0001; genotype x sex "F (1, 224) = 113.3", p < 0.0001
	**CA3**	3-way ANOVA	layer "F (3, 224) = 63.22", p < 0.0001; genotype "F (1, 224) = 19.94", p = 0.0001; sex "F (1, 224) = 45.02", p < 0.0001; genotype x sex "F (1, 224) = 76.18", p < 0.0001

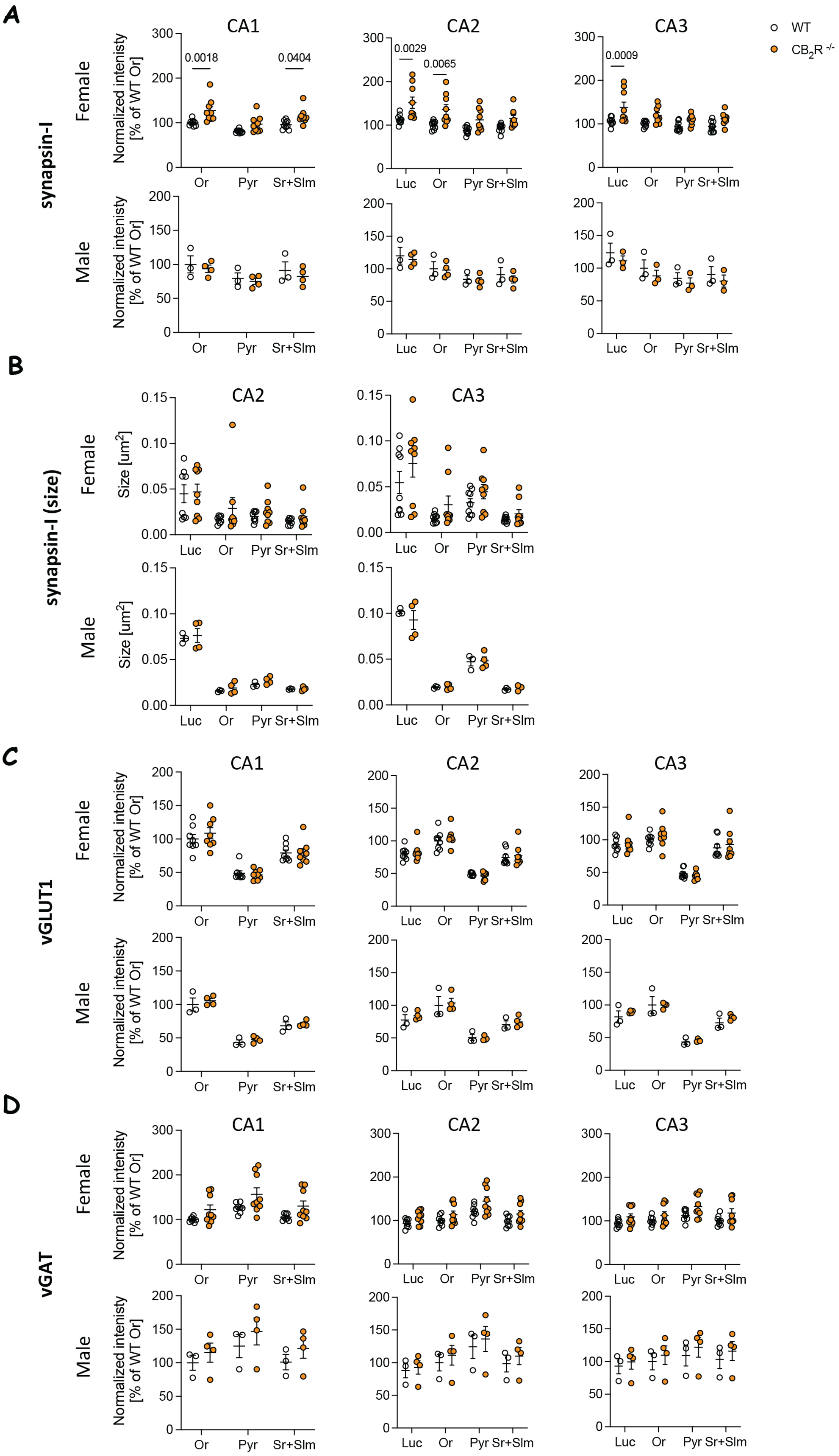
A
synapsin-I
Female
Male
CA1
CA2
CA3
WT
$CB_2R^{-/-}$
Normalized intenisty [% of WT Or]
0.0018
0.0404
0.0029
0.0065
0.0009
Luc
Or
Pyr
Sr+Slm
B
synapsin-I (size)
Size [um^2]
C
vGLUT1
D
vGAT

Supplement Figure S1. Mean per animal values for all presented data in Figures 1-4. (**A**) Figure 1: synapsin-I (green) (**B**) Figure 2: average size of synapsin-I particles; (**C**) Figure 3: vGLUT1; (**D**) Figure 4: vGAT. Or - stratum oriens; Luc – stratum lucidum; Pyr – stratum pyramidale; Sr+Slm – stratum radiatum and stratum lacunosum-moleculare. White circle – WT; orange circle – $CB_2R^{-/-}$ mice. Each datapoint represents one mouse. Upper panel – female mice (WT: N = 8-9 mice; $CB_2R^{-/-}$: N = 9 mice); right panel - male mice (WT: N = 3 mice; $CB_2R^{-/-}$: N = 4 mice). Left panel – CA1 region; middle panel – CA2 region; right panel – CA3 region. Data is displayed as mean value ± SEM. Data was analyzed by two-way ANOVA followed by Sidak's multiple comparison test. The exact p-values are indicated on the graph and reported in Supplementary Table S3.

Supplementary Table S3. Detailed results from 2-way ANOVA analysis for Supplement Figure S2.

		Statistical test		Posthoc test	
Figure	Panel	Test name	result	Test name	result
S2. Mean per animal	**a:** females: CA1	2-way ANOVA	layer "F (2, 48) = 11.77", p < 0.0001; genotype "F (1, 48) = 23.39", p < 0.0001	Šídák's multiple comparisons test	Or p = 0.0018; Sr+Slm p = 0.0404
	**a:** females: CA2	2-way ANOVA	layer "F (3, 64) = 7.389", p = 0.0003; genotype "F (1, 64) = 30.75", p < 0.0001	Šídák's multiple comparisons test	Luc p = 0.0029; Or p = 0.0065
	**a:** females: CA3	2-way ANOVA	layer "F (3, 64) = 5.153", p = 0.0030; genotype "F (1, 64) = 27.28", p < 0.0001	Šídák's multiple comparisons test	Luc p = 0.0009
	**a:** males: CA2	2-way ANOVA	layer "F (3, 20) = 8.041", p = 0.0010		
	**a:** males: CA3	2-way ANOVA	layer "F (3, 16) = 4.876", p = 0.0135		
	**b:** females: CA2	2-way ANOVA	layer "F (3, 60) = 7.020", p = 0.0004		
	**b:** females: CA3	2-way ANOVA	layer "F (3, 64) = 13.05", p < 0.0001; genotype "F (1, 64) = 5.207", p = 0.0258		
	**b:** males: CA2	2-way ANOVA	layer "F (3, 20) = 106.7", p < 0.0001		
	**b:** males: CA3	2-way ANOVA	layer "F (3, 20) = 111.0", p < 0.0001		
	**c:** females: CA1	2-way ANOVA	layer "F (2, 42) = 49.37", p < 0.0001		
	**c:** females: CA2	2-way ANOVA	layer "F (3, 56) = 58.52", p < 0.0001		
	**c:** females: CA3	2-way ANOVA	layer "F (3, 56) = 45.78", p < 0.0001		
	**c:** males: CA1	2-way ANOVA	layer "F (2, 15) = 77.35", p < 0.0001		
	**c:** males: CA2	2-way ANOVA	layer "F (3, 20) = 22.92", p < 0.0001		
	**c:** males: CA3	2-way ANOVA	layer "F (3, 20) = 38.20", p < 0.0001		
	**d:** females: CA1	2-way ANOVA	layer "F (2, 45) = 5.779", p = 0.0058; genotype "F (1, 45) = 11.39", p = 0.0015		
	**d:** females: CA2	2-way ANOVA	layer "F (3, 60) = 8.730", p < 0.0001; genotype "F (1, 60) = 12.57", p = 0.0008	Šídák's multiple comparisons test	Pyr p = 0.0632
	**d:** females: CA3	2-way ANOVA	layer "F (3, 60) = 3.615", p = 0.0181; genotype "F (1, 60) = 11.51", p = 0.0012		

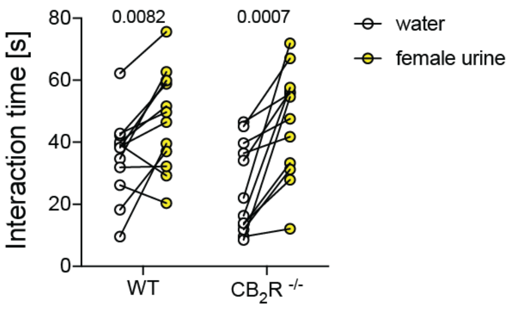

Supplement Figure S2. $CB_2R^{-/-}$ have an intact olfaction. Interaction time of mice with water-stained (white circle) or female urine-stained (yellow circle) cotton swab. Increase in the interaction time suggests lack of olfactory deficits. Circles represent data from individual mice (N = 12 mice/genotype). Data was analyzed separately for each genotype by paired t-test. The exact p-values are indicated on the graph.

Supplementary Materials and Methods

Olfaction test protocol was modified from "Simple Behavioral Assessment of Mouse Olfaction"; The Olfactory Habituation/Dishabituation Test (Yang & Crawley, 2009). The test was performed only on the male group after the partner recognition test. A cotton swab was inserted from the top of the cage around 2 - 3 cm from the grid top. In the first trial, 10 µl of water was placed on the cotton swab and for 2 minutes interaction time of the mouse with a cotton swab was recorded. Then the cotton swab was removed from the cage for 1 minute and another cotton swab was inserted with a 10 µl drop of female urine. Once again, the interaction time was recorded for 2 minutes. Proper sense of smell would cause no change or an increase in interaction time in the second trial. Decrease of interaction time would suggest a habituation to the cotton swab and olfactory disfunction.

Bibliography

Yang, M., & Crawley, J. N. (2009). Simple Behavioral Assessment of Mouse Olfaction Mouse Olfaction. *Current Protocols in Neuroscience*, (CHAPTER: Unit–8.24), 1–14. https://doi.org/10.1002/0471142301
